# Association Between the Blood Urea Nitrogen-to-Creatinine Ratio Trajectories and Clinical Outcomes in Critically Ill Hemorrhagic Stroke Patients: Insights from MIMIC-IV Database

**DOI:** 10.3390/jcm14228141

**Published:** 2025-11-17

**Authors:** Xinyuejia Huang, Huixuan Luo, Hao Deng, Yang Wu, Mengqi Wang, Linglong Xiao, Xiaoman Shi, Wei Pan, Yuan Gao, Wei Wang

**Affiliations:** Department of Neurosurgery, West China Hospital of Sichuan University, Chengdu 610041, China; hxyj2023@stu.scu.edu.cn (X.H.); ananova16@163.com (H.L.); 2023324025260@stu.scu.edu.cn (H.D.); wuyang1@stu.scu.edu.cn (Y.W.); wmq_1101@scu.edu.cn (M.W.); xll2021@stu.scu.edu.cn (L.X.); 2023224025415@stu.scu.edu.cn (X.S.); 18011376726@163.com (W.P.); gaoyuan_scu@wchscu.cn (Y.G.)

**Keywords:** urea nitrogen-to-creatinine ratio, hemorrhagic stroke, MIMIC-IV database, all-cause mortality, prognosis

## Abstract

**Background:** Hemorrhagic stroke (HS) accounts for approximately 30% of all stroke cases and has high mortality in the intensive care unit (ICU). The blood urea nitrogen-to-creatinine ratio (BUNCR) is a potential biomarker of catabolic stress in critically ill patients. Meanwhile, its dynamic prognostic value in ICU-admitted HS patients remains unclear. This study utilized Group-based Trajectory Modeling (GBTM) to investigate associations between early BUNCR patterns and mortality. **Methods:** This study was conducted using data from the MIMIC-IV (v2.2) database. HS cases were identified via ICD-9/10. BUNCR trajectories were assessed by applying GBTM during the first 7 days. Outcomes were all-cause mortality (ACM) on day 28, on day 90, and at 1 year, with ICU and in-hospital mortality also evaluated. Kaplan–Meier survival curves and log-rank test compared survival across groups. Multivariable Cox proportional models adjusted for confounders and subgroup analysis assessed robustness. **Results:** Among 2559 patients (52.48% male), mortality was 10.16% (ICU), 14.07% (in-hospital), 17.00% (28-day), 22.43% (90-day), and 36.97% (1-year). Three BUNCR trajectories were identified: Group 1 (upward–downward, *n* = 655), Group 2 (stable upward, *n* = 1270), and Group 3 (downward–upward, *n* = 634). Group 2 had the highest ACM risk at 28-day, 90-day, and 1-year (*p* < 0.01), and was identified as a significant risk factor in multivariate Cox regression. Subgroup revealed significant interactions of BUNCR trajectories with age and sepsis. **Conclusions**: Distinct BUNCR trajectories were significantly associated with ACM in critically ill HS patients. Persistently increasing BUNCR predicted the poorest outcomes, underscoring its potential as a dynamic biomarker for timely risk stratification and informed ICU decisions.

## 1. Introduction

Stroke is the second leading cause of death worldwide, following ischemic heart disease and the COVID-19 pandemic in 2021, and remains one of the most fatal and disabling diseases globally [1]. Hemorrhagic stroke including intracerebral hemorrhage (ICH) and spontaneous subarachnoid hemorrhage is a life-threatening condition with a poor prognosis [2]. As the global population continues to age, the burden of HS, particularly among critically ill patients in ICU, is increasing significantly. Identifying reliable prognostic markers is of vital importance for predicting adverse outcomes in HS patients admitted to the ICU. Several biomarkers have shown potential prognostic value in critically ill HS patients. For example, Chen et al. [3] reported that an elevated blood urea nitrogen-to-creatinine ratio (BUNCR) on the first day of ICU admission was associated with poor short-term outcomes. Similarly, an increased triglyceride-glucose index (TyG-i) on admission has been linked to higher all-cause mortality in HS patients [4]. Furthermore, low levels of the hemoglobin-to-red cell distribution width ratio (HRR) have been associated with heightened mortality in subarachnoid hemorrhage cases [5]. However, most of these studies have only focused on static values measured at a single time point, often early in the ICU stay, and have primarily investigated short-term outcomes. The prognostic value of dynamic changes in these predictors and their association with long-term mortality is unclear. Understanding these longitudinal patterns may provide deeper insights into the progression of HS and inform individualized treatment strategies.

Blood urea nitrogen (BUN) and creatinine are metabolic end products of protein degradation and are widely utilized to measure kidney function [6,7]. It has been proposed as a surrogate marker of both muscle catabolism and renal function, and may serve as a potential biomarker in persistent critical illness [8,9]. Previous studies have reported that elevated BUNCR levels are characteristic of patients with prolonged critical illness and extensive skeletal muscle wasting, commonly observed in ICU populations [8,10].

It may be crucial for us to detect ongoing catabolic processes and predict clinical outcomes among them [7,11]. However, most existing studies have examined BUNCR at a single time point, such as upon ICU admission. It remains unclear whether BUNCR is merely a marker of poor long-term prognosis or a modifiable target for therapeutic intervention [12,13]. Therefore, we hypothesized that dynamic changes in BUNCR are associated with the prognosis of patients with HS during their ICU stay.

This study utilized data from the Medical Information Mart for Intensive Care-IV (MIMIC-IV, version 2.0) database to examine the association between BUNCR trajectories during the first week of ICU admission and all-cause mortality at 28 days, 90 days, and one year in critically ill patients with HS.

## 2. Methods

### 2.1. Acknowledgements Statement

During the preparation of this manuscript, the authors declare that Gen AI was used in the creation of this manuscript. This manuscript was polished after the author finished writing with the assistance of an AI chatbot, ChatGPT (Version: GPT-4, Model: GPT-4-turbo, Source: OpenAI). Specific queries were structured in natural language, focusing on the improvement of sentence structure, conciseness, and academic tone, without altering the scientific content or interpretation of the findings. The authors have reviewed and edited the output and take full responsibility for the content of this publication.

### 2.2. Patients and Data Collection

Patient information was sourced from the Medical Information Mart for Intensive Care IV (MIMIC-IV, version 2.0) database [14]. Extracted data included patient demographics, vital signs, comorbidities, laboratory test results, medication records, and survival outcomes. Data extraction was performed by an author, Hao Deng (Record ID: 66213243) who completed the required training to access the MIMIC-IV database and adhered to the data use agreement. Before the formal data extraction, standardized extraction protocols were developed and pilot-tested to ensure clarity, feasibility, and reproducibility. Multiple validation measures were employed to ensure data accuracy, including independent verification of critical variables and automated statistical checks to uncover and amend potential input mistakes or discrepancies.

HS cases were extracted from the MIMIC-IV database using the International Classification of Diseases (ICD)-9/10 diagnostic codes. Specifically, we used ICD-9 code 431 and ICD-10 codes I610–I619 and I62.9 for intracerebral hemorrhage (ICH), and ICD-9 code 430 along with ICD-10 codes I60, I600–I6012, I6000–I6002, I6020–I6022, I6030–I6032, and I6050–I6052 for non-traumatic HS. The following exclusion criteria were applied to ensure data quality and integrity: (1) individuals younger than 18 years at initial admission; (2) repeated ICU admissions for HS, with only the first episode retained; (3) ICU length of stay less than 48 h; (4) absence of complete baseline information from the first ICU stay.

After screening 364,627 participants, we included 4059 those diagnosed with HS. Among them, 2559 patients with complete data and who met all eligibility criteria were included in the final analysis. Data extraction was performed using PostgreSQL (version 13.7.2) and Navicat Premium (version 16). Structured Query Language (SQL) scripts were used to retrieve relevant variables from the database. Laboratory data and severity assessments were collected during the initial 24 h following ICU stay, and variables were categorized into five domains: (1) Demographics encompassing age, gender, ethnicity and marital status. (2) Vital signs, including, heart rate, respiratory rate, systolic blood pressure (SBP), diastolic blood pressure (DBP) and mean blood pressure (MBP), body temperature (℃), and oxygen saturation measured via pulse oximetry (SpO2). (3) Severity scores: Sequential Organ Failure Assessment (SOFA) score, Acute Physiology III score (APS-III), Systemic Inflammatory Response Syndrome (SIRS) score, Simplified Acute Physiology Score (SAPS)-II, Oxford Acute Severity of Illness Score (OASIS) and Glasgow Coma Scale (GCS). (4) Laboratory parameters: hemoglobin (Hb), red blood cell count (RBC), white blood cell count (WBC), platelet count (PLT), activated partial thromboplastin time (APTT), prothrombin time (PT), serum creatinine, blood urea nitrogen (BUN) and blood urea nitrogen-to-creatinine ratio (BUNCR). (5) Comorbidities and treatments: Charlson Comorbidity Index (CCI), sepsis, peripheral vascular disease (PVD), intraventricular hemorrhage (IVH), hypertension, Liver cirrhosis, Chronic-kidney disease (CKD), malignant tumors, type 2 diabetes mellitus (T2DM), type 1 diabetes mellitus (T1DM), heart failure, coronary heart disease (CHD), and chronic obstructive pulmonary disease (COPD). Treatment-related interventions covered mechanical ventilation (MV) and antidiuretic hormone therapy.

### 2.3. Group-Based Trajectory Models

In the study, creatinine and serum urea nitrogen from day 1 to day 7 were collected to calculate the BUNCR for each patient. A group-based trajectory model (GBTM) was constructed using the hlme() function from the lcmm package in R (Version: 4.1.3). To eliminate inter-individual differences in absolute values and emphasize relative trends, BUNCR values were standardized using Z-score normalization within each patient. We applied natural cubic splines with 3 degrees of freedom to flexibly model the non-linear time-dependent trends of BUNCR. To determine the optimal number of BUNCR trajectories, we fitted trajectory models with two to five latent classes. The stable-upward trajectory pattern consistently appeared across the three-, four- and five-class models, and patients within this pattern were highly consistent across models. Although the Bayesian Information Criterion (BIC) of the three-class model was not the lowest (Table S4), models with a higher number of classes yielded smaller subgroup sample sizes and overly complex trajectory patterns, which reduced clinical interpretability. Furthermore, the stable-upward group in the three, four- and five-class models was associated with a significantly higher risk of all-cause mortality, while no other trajectory groups showed significant survival differences (Figure S4). Considering both statistical fit and clinical interpretability, the three-class model was selected as the final model for subsequent analyses. Patients were assigned to one of the three BUNCR trajectory groups according to their maximum posterior probability: Group 1 (G1): BUNCR rapidly increased from a low to a high level, then declined. Group 2 (G2): BUNCR steadily increased from a low to a high level over time. Group 3 (G3): BUNCR rapidly decreased from a high to a low level, then back up.

The appropriate count of trajectory groupings was determined based on combined standards: (1) Bayesian Information Criterion (BIC), which prioritizes values close to zero to signal enhanced model fit. (2) Group size requirement: Each trajectory group was required to contain more than 5% of the study population to preserve both relevance and statistical robustness. (3) Visual discernibility: The trajectory paths had to be clearly distinguishable, confirming the practical distinction among groups.

### 2.4. Clinical Outcomes

The primary clinical endpoints were 28-day, 90-day and 1-year all-cause mortality. The in-hospital mortality was assessed in patients who died during the initial hospitalization, and the ICU mortality was assessed in patients who died during their ICU stay. The 28-day, 90-day and 1-year mortality outcomes were defined, respectively, as all-cause death within 28 days, 90 days and one year of ICU admission.

### 2.5. Statistical Analysis

Statistical analyses and plot making were performed with R 4.1.3 software (https://cran.r-project.org/bin/windows/base/old/4.1.3/, accessed on 11 June 2025). Statistical significance was determined using a two-tailed *p*-value of < 0.05. Categorical variables were summarized as frequencies and percentages, and differences between BUNCR trajectory groups were assessed using the Chi-square test. Missing data for covariates were imputed using the “missForest” package.

Kaplan–Meier (K-M) survival analysis was conducted to determine the prognostic impact of BUNCR trajectory groups on 28-day, 90-day and 1-year all-cause mortality after ICU admission. Differences in survival distributions among groups were assessed using the log-rank test. If K-M survival curves showed evidence of crossing, we performed a landmark analysis to evaluate potential time-dependent effects. The landmark time point was determined based on visual inspection of the survival curves and clinical relevance. Landmark analyses were implemented in R using the “jskm” package. To further evaluate the association between BUNCR trajectories and mortality, we used multivariate Cox proportional hazards models and adjusted covariates, including age, gender, race, hypertension, liver cirrhosis, CKD, diabetes mellitus, IVH, with results reported as hazard ratio (HR) and 95% confidence interval (CI).

Several subgroup analyses were performed to examine potential factors influencing the relationship between BUNCR trajectory groups and outcomes in HS patients, including gender (male vs. female), age (<65 years vs. ≥65 years) and comorbidities. We included an interaction term between BUNCR group and stratification covariates in the model to test for potential effect modification in the fully adjusted model. In the sensitivity analysis, we fitted trajectory models with two to five latent classes to explore the robustness of our findings across different trajectory groupings. We compared the trajectory characteristics across the models and performed landmark analysis on those models with similar trajectory features to determine whether groups with similar trajectories corresponded to similar prognostic outcomes. Additionally, we further adjusted for the occurrence of craniotomy and continuous renal replacement therapy (CRRT) in a multivariable Cox regression model to account for potential confounding factors.

## 3. Results

### 3.1. BUNCR Trajectories and Baseline Characteristics

**Figure 1:**
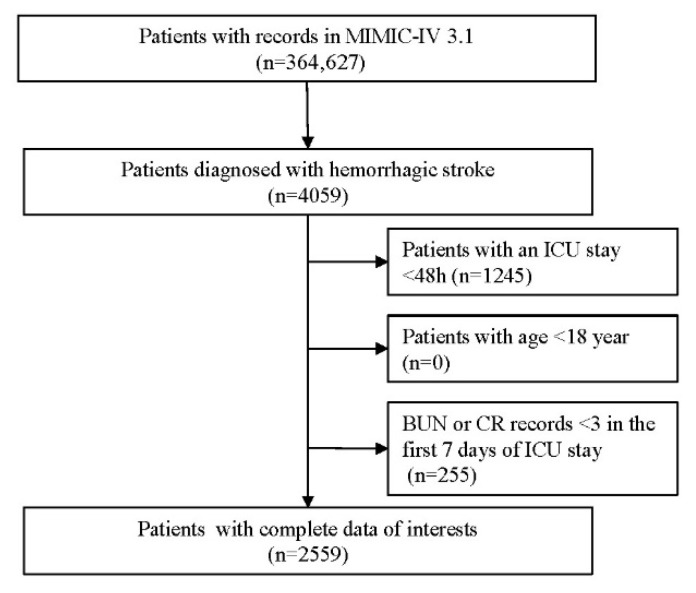
Flow diagram of participants selection.

**Figure 2:**
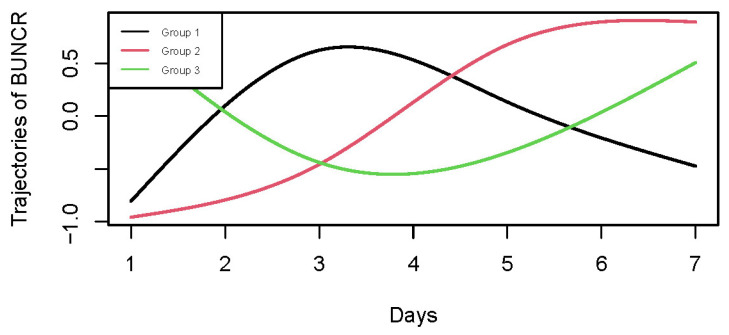
Group-based trajectory modeling of BUNCR in hemorrhagic stroke patients during the first 7 days of ICU admission. GBTM identified three distinct BUNCR trajectory patterns: G1 (upward–downward), G2 (stable-upward), and G3 (downward–upward).

A total of 4059 patients diagnosed with HS were initially identified from the MIMIC-IV database. Following application of the predefined inclusion and exclusion criteria, 2559 ICU patients with first-time HS admissions were retained for the final analysis. The selection procedure is shown in Figure 1, and the baseline characteristics of the cohort stratified by BUNCR trajectory groups are summarized in Table 1. The mean (±SD) age of the study population was 65.36 ± 15.61 years, with 1216 females (47.52%) and 1343 males (52.48%). Based on BUNCR trends over the first 7 days of ICU admission, all patients were categorized into three distinct trajectory groups (Figure 2): Group 1 (G1, upward–downward): BUNCR increases rapidly from a low to a high level and then declined; Group 2 (G2, stable upward): BUNCR gradually increases from a low to a high level over time; Group 3 (G3, downward–upward): BUNCR decreases rapidly from a high to a low level over time and then returned to elevated levels. The temporal trends of BUNCR for these three groups are presented in Figure 2.

Statistically significant differences were observed among the 3 groups in terms of age, SBP, all severity scores, laboratory parameters (including Hb, RBC, BUN and BUNCR), comorbidities (including CCI, Sepsis, Hypertension and Liver cirrhosis), treatment (including MV), and ACM of 28-day, 90-day and 1-year (all *p* < 0.01). Notably, the initial BUNCR values recorded within the first 24 h of ICU admission had a mean value of 18.92 ± 7.95, with significant variation across trajectory groups (*p* < 0.01). The G2 presented a lowest BUNCR level (17.56 ± 6.98) compared to other groups.

### 3.2. Association Between BUNCR Trajectories and Clinical Outcomes

**Figure 3:**
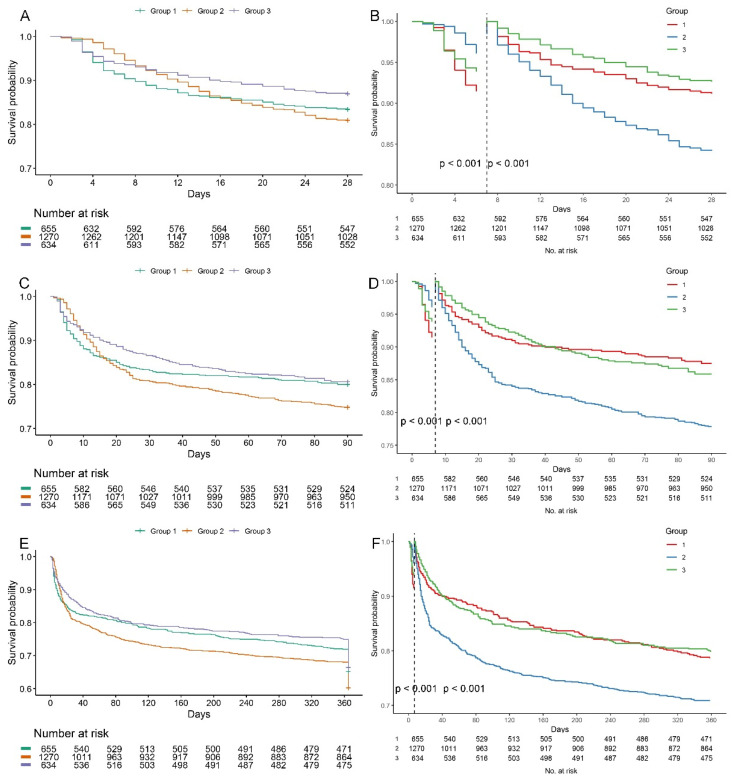
Kaplan–Meier survival curves according to BUNCR trajectory groups. Survival probabilities at 28 days (**A**), 90 days (**C**), and 1 year (**E**) after ICU admission are shown for each BUNCR trajectory group. Statistical differences were assessed using the log-rank test; all pairwise comparisons showed *p* < 0.001. (**B**,**D**,**F**) Seven-day landmark analyses were performed to account for early curve crossing and demonstrated significantly reduced survival probabilities in Group 2 beginning at day 7 after ICU admission (*p* < 0.05, log-rank test).

The KM survival curves of the three trajectory groups were listed in Figure 3A,C,E. It was observed that G2 exhibited a decreased survival probability at 28 days, 90 days, and one year, uncovering substantial variances in mortality rates among the three BUNCR trajectory groups. However, the survival curve of G2 crossed with that of other groups. Therefore, we conducted landmark analyses which showed a significantly lower survival probabilities in G2 7 days after ICU admission (*p* < 0.05) (Figure 3B,D,F).

Multivariate Cox proportional hazards analysis further confirmed these findings (Table 2). Using G2 as the reference, the HR for 28-day ACM was 0.91 (95% CI: 0.72–1.14, *p* = 0.421) for G1 and 0.73 (95% CI: 0.57–0.94, *p* = 0.014) for G3, indicating a significantly reduced short-term mortality risk in G3. At 90 days, both G1 and G3 showed a 20% lower mortality risk compared to G2 (HR = 0.80, *p* < 0.05 for both). Similarly, for 1-year ACM, G1 (HR = 0.83, 95% CI: 0.71–0.97, *p* = 0.021) and G3 (HR = 0.84, 95% CI: 0.72–0.99, *p* = 0.037) maintained a lower risk. In contrast, ICU mortality was not significantly associated with BUNCR trajectories in either univariate or multivariate analyses. Similarly, no statistically significant differences in in-hospital mortality were observed across the three groups (Table 1). These results underscore that persistently rising BUNCR levels (G2) are independently associated with the worst prognosis, particularly in long-term outcomes.

### 3.3. Subgroup Analysis and Sensitivity Analysis

For 28-day ACM, patients in G3 (vs. G2) exhibited significantly lower mortality risk among several subgroups, notably among individuals aged over 65 years (HR: 0.67; 95% CI: 0.49–0.93), females (HR: 0.58; 95% CI: 0.4–0.86), white patients (HR: 0.61, 95%CI: 0.42–0.91), patients with liver cirrhosis (HR: 0.69, 95%CI: 0.53–0.91) and individuals not afflicted by sepsis (HR: 0.61; 95% CI: 0.45–0.82) (Figure S5A). Additionally, a significant interaction was observed with sepsis (G3 vs. G2, *p* = 0.04). In 90-day ACM, the reduced mortality risk of G3 was statistically significant in patients without sepsis (HR: 0.72; 95% CI: 0.56–0.91) (Figure S5B). For 1-year ACM, the survival benefit of G3 remained evident among individuals younger than 65 years (HR: 0.76; 95% CI: 0.62–0.93) and those without sepsis (HR: 0.78; 95% CI: 0.65–0.93) (Figure S5C). There was a significant interaction between BUNCR trajectory groups and age (G3 vs. G2, *p* = 0.02).

In the sensitivity analysis, after further adjustment for craniotomy and CRRT, the G2 trajectory still corresponded to a significantly increased risk of all-cause mortality (Tables S5 and S6). We observed the presence of the stable-upward trajectory in both the four-trajectory and five-trajectory models (Figures S1–S3). Notably, the patient populations assigned to this trajectory were highly consistent across the different models (Tables S1–S3). Furthermore, patients with the stable-upward trajectory consistently exhibited a higher risk of mortality (Figure S4).

## 4. Discussion

Our study analyzed 2559 patients with hemorrhagic stroke admitted to the ICU, collected from the MIMIC-IV database and used GBTM to characterize the dynamic trajectories of BUNCR during the first week of ICU admission, which is first to explore the association between dynamic BUNCR variations and survival probabilities in critically ill HS patients. And it demonstrates several new insights. Initially, three distinct BUNCR trajectory groups were identified: G1, upward–downward; G2, stable upward; G3, downward–upward. Our findings demonstrated that these BUNCR trajectories were significantly associated with differences in survival probability. In particular, G2 was associated with the highest risk of all-cause mortality across all time points, compared to the other two groups. This indicated that the progressive increase in BUNCR levels over the first 7 days of ICU admission was strongly indicative of poor prognosis.

The identification of three distinct BUNCR trajectories in this study underscores the heterogeneity in metabolic responses among HS patients in the ICU. These trajectories suggest that variations in catabolic state and renal function over time may contribute to differences in clinical outcomes. Notably, our findings indicate that most patients experienced unstable BUNCR levels during the first 7 days of ICU admission. Among them, G2, characterized by a persistently increasing trend from low to high BUNCR, demonstrated the worst prognosis, despite relatively modest absolute fluctuations. A previous study identified BUNCR as a significant risk factor of mortality in patients with non-traumatic intracranial hemorrhage. Higher BUNCR value at admission corresponded to a significant higher mortality rate at 30 day (HR: 1.03, 95CI: 1.02–1.04, *p* < 0.001) and 1 year (HR: 1.05, 95CI: 1.04–1.06, *p* < 0.001) [3]. Conversely, in our study, G2 had the lowest BUNCR values at ICU admission but also showed highest mortality rates at different time-points. This suggests that the directional trend of BUNCR, rather than isolated numerical values, may carry stronger prognostic significance.

Previous studies have proposed that elevated BUNCR levels may reflect a combination of enhanced endogenous catabolism and excess protein or amino acid load, both of which are commonly encountered in critically ill patients [15,16]. Moreover, some investigations have used either BUN or serum creatinine as independent predictors of mortality in cerebrovascular or stroke ICU population [17]. Muscle wasting, a hallmark of persistent critical illness, has been closely associated with prolonged ICU stays and increased mortality [10]. Studies have reported a rapid and continuous decline in serum creatinine during the first 4 days of ICU admission, likely due to reduced muscle mass and altered metabolism [18]. At the same time, persistent muscle catabolism and the subsequent liberation of amino acids have been linked to elevated BUN levels, reflecting ongoing nitrogen load throughout the ICU stay [8,19]. However, they did not validate this relationship in critically ill HS cohorts. In our analysis, the persistently increasing BUNCR trajectory (G2) bore a strong association with ACM at 28 days, 90 days, and 1 year, while fluctuating trajectories (G1/3) showed significantly lower adjusted hazards. Our study suggests that it is the trend of BUNCR change, rather than a single value, which more accurately mirrors the sustained catabolic and metabolic status of HS patients in the ICU. BUN itself is a derivative measure of blood nitrogen content in the form of urea, calculated as BUN (mg/dL) × 2.142 = urea (mg/dL) [20]. In healthy individuals with an average protein intake, stable volume status, and electrolytes balance, BUNCR is typically around 10:1 [21,22]. Deviations from this ratio in the ICU setting may therefore reflect not only the severity of cerebral injury but also metabolic disturbances relevant to patient prognosis.

In hemorrhagic stroke, acute elevations in intracranial pressure and activation of neuroendocrine stress pathways trigger a surge in circulating catecholamines, cortisol, and antidiuretic hormone, leading to decreased renal perfusion, reduced glomerular filtration rate (GFR), and consequent renal impairment [23]. These changes result in excessive production and accumulation of BUN. Furthermore, enzymatic degradation of hematomas releases amino acids that are converted to ammonia upon entering the systemic circulation. The subsequent hepatic conversion of ammonia to urea further increases BUN levels. This inverse trend between urea and creatinine contributes to elevated BUNCR [24]. Several studies in critically ill patients have also shown that an increased BUNCR reflects a hypercatabolic phenotype associated with higher mortality [25,26,27]. Therefore, a persistently rising BUNCR trajectory (G2) likely reflects sustained catabolic stress, renal hypoperfusion, and systemic inflammation. These systemic disturbances exacerbate metabolic instability, impair cerebrovascular autoregulation, and promote secondary organ dysfunction, ultimately leading to worse neurological outcomes and higher mortality in HS patients [28]. In contrast, fluctuating or recovering BUNCR trajectories (as observed in G1 and 3) may indicate partial recovery of renal and metabolic homeostasis, corresponding to improved survival. This proposed pathophysiological framework supports the prognostic relevance of dynamic BUNCR monitoring in critically ill HS patients and aligns with our findings.

As previously reported, BUN primarily originates from the catabolism of endogenous proteins, and its plasma concentration is determined by four main factors: protein intake, the rates of amino acid anabolism and catabolism, and the hepatic capacity for urea production [22]. BUN is freely filtered by the glomerulus and subsequently reabsorbed in the renal tubules, making it highly sensitive to changes in renal function [29]. Importantly, plasma BUN levels depend not only on the GFR but also on tubular reabsorption, which is modulated by neurohormonal activity, including activation of the renin–angiotensin–aldosterone system (RAAS) activity [30]. Urea reabsorption primarily occurs in the proximal tubules, with additional reabsorption in the collecting ducts under the influence of vasopressin [31]. Various clinical conditions, such as gastrointestinal bleeding, high-protein intake, and extensive tissue breakdown, can also increase nitrogen metabolism and result in elevated BUN levels [32,33]. Serum creatinine (SCr), in contrast, is a byproduct of muscle metabolism and is one of the most widely used biomarkers to estimate renal function, as it correlates closely with GFR [34]. Since creatinine is largely derived from skeletal muscle, muscle wasting leads to reduced SCr production [35], the level of which is also often used to reflect the constantly breaking-down status of skeletal muscles. Although creatinine is freely filtered by the glomerulus, a small portion is also secreted by renal tubules, which may lead to a tiny bias in the appraisal of GFR when using creatinine-based calculations [29]. Our findings may also reflect these relationships, with persistently elevated BUNCR trajectory (G2) potentially mirroring the combined effects of sustained urea production, reduced creatinine production, and possible amplification by tubular reabsorption mechanisms. These combined physiological dynamics underscore the importance of interpreting BUN and creatinine in tandem, particularly through the BUN-to-creatinine ratio (BUNCR), which may offer better insight into the balance between renal filtration and catabolic stress in ICU patients.

This investigation has several strengths. First, to our knowledge, this is the first investigation to examine the relationship between longitudinal BUNCR trajectories and all-cause mortality in critically ill HS patients, using a real-world American ICU database. Second, we demonstrated that persistently elevated BUNCR trajectory is an independent predictor of poor prognosis, reinforcing its potential utility as a dynamic biomarker in the ICU setting. Nonetheless, there are some limitations in the study. Although we employed multivariable adjustments and subgroup analyses to reduce confounding, residual confounding from unmeasured variables cannot be ruled out. For instance, the MIMIC-IV database lacks detailed clinical information such as eliminations based on the National Institutes of Health Stroke Scale, stroke occurrence timing, or precise mortality causes. The lack of detailed information on cause-specific mortality prevents us from analyzing the impact of specific causes of death on our findings. For hemorrhagic stroke, neurological death is often related to intracranial hypertension and brain herniation, typically occurring during the acute phase of bleeding, while deaths after the acute phase are usually attributed to non-neurological complications. Although we observed an association between the stable-upward BUNCR trajectory and increased all-cause mortality risk, we hypothesize that this trajectory may be more closely linked to systemic causes of death. Future studies with access to cause-specific mortality data are needed to validate this hypothesis. In addition, at least three repeating BUNCR measurements were necessary to ensure the robustness and interpretability of the trajectory model, which may introduce selection bias, as patients with fewer laboratory tests—often those with early discharge, rapid recovery, or early death—were excluded. The identified trajectories may not fully capture the variability of BUNCR dynamics across the entire population of patients with hemorrhagic stroke, thereby limiting the external validity of our findings. Furthermore, this analysis was based on a single-center ICU database, which may limit the generalizability of our findings and introduce selection bias, given that ICU patients typically present with more severe conditions. In future studies, it is essential to conduct multicenter, prospective investigations incorporating broader patient populations and more granular clinical data. Such studies will be crucial to validating our results and exploring the clinical applicability of BUNCR trajectory monitoring as a predictive tool in hemorrhagic stroke management.

## 5. Conclusions

In our study, we identified three distinct BUNCR trajectory groups among critically ill patients with HS. These trajectories were significantly associated with ACM, suggesting that dynamic changes in BUNCR levels may serve as a valuable prognostic biomarker in ICU. Notably, patients with a persistently elevated BUNCR during the initial seven days of ICU stay experienced the highest risk of mortality. This finding highlights the usefulness of BUNCR trajectory monitoring in guiding clinical risk assessment and management decisions. By integrating BUNCR trajectories into early assessment protocols, clinicians may be able to identify high-risk patients more promptly and tailor therapeutic strategies accordingly. Nevertheless, multicenter and prospective studies are needed to further verify these findings.

## Figures and Tables

**Table 1:** Baseline characteristics and outcomes of included participants.

Variables	Overall (*n* = 2559)	G1 (*n* = 655)	G2 (*n* = 1270)	G3 (*n* = 634)	^a^ *p*
Age	65.36 ± 15.61	65.6 ± 15.51	66.27 ± 15.54	63.3 ± 15.68	<0.01
**Gender** (*n*, %)					
Female	1216 (47.52)	300 (45.80)	601 (47.32)	315 (49.68)	0.37
Male	1343 (52.48)	355 (54.20)	669 (52.68)	319 (50.32)	
**Race** (*n*, %)					
White	249 (9.73)	57 (8.70)	128 (10.08)	64 (10.09)	0.05
Black	100 (3.91)	31 (4.73)	46 (3.62)	23 (3.63)	
Mexican American	755 (29.50)	170 (25.95)	409 (32.20)	176 (27.76)	
Other	1455 (56.86)	397 (60.61)	687 (54.09)	371 (58.52)	
**Marital status** (*n*, %)					
Divorced	172 (6.72)	39 (5.95)	85 (6.69)	48 (7.57)	0.15
Married	1504 (58.77)	378 (57.71)	763 (60.08)	363 (57.26)	
Single	639 (24.97)	174 (26.56)	291 (22.91)	174 (27.44)	
Widowed	244 (9.53)	64 (9.77)	131 (10.31)	49 (7.73)	
**Vital signs**					
Heart rate (beats/min)	82.88 ± 17.54	83.05 ± 16.96	82.6 ± 17.49	83.25 ± 18.23	0.71
SBP	133.26 ± 22.53	133.06 ± 20.77	134.9 ± 23.16	130.17 ± 22.71	<0.01
DBP	74.93 ± 17.9	74.39 ± 16.99	75.65 ± 18.76	74.03 ± 16.98	0.12
MBP	90.04 ± 17.72	89.96 ± 17.23	90.78 ± 18.39	88.61 ± 16.78	0.04
Respiratory rate (times/min)	18.65 ± 5.47	18.73 ± 5.68	18.62 ± 5.4	18.62 ± 5.4	0.91
SpO_2_	97.4 ± 3.65	97.24 ± 2.77	97.54 ± 4.24	97.29 ± 3.12	0.15
Temperature (°C)	98.34 ± 1.8	98.43 ± 1	98.35 ± 1.63	98.25 ± 2.59	0.23
**Severity**					
SOFA	3.52 ± 2.81	3.2 ± 2.55	3.61 ± 2.69	3.68 ± 3.25	<0.01
APS-III	38.22 ± 17.8	35.73 ± 16.29	38.65 ± 17.59	39.95 ± 19.4	<0.01
SIRS	2.39 ± 0.98	2.33 ± 0.99	2.47 ± 0.97	2.3 ± 0.98	<0.01
SAPS-II	32.95 ± 12.05	31.93 ± 11.81	33.83 ± 11.73	32.25 ± 12.81	<0.01
OASIS	31.18 ± 7.93	29.97 ± 7.81	32.23 ± 7.7	30.32 ± 8.22	<0.01
GCS	12.65 ± 3.06	12.98 ± 2.75	12.39 ± 3.25	12.85 ± 2.94	<0.01
**Laboratory parameters**					
Hb (g/L)	11.92 ± 1.99	12.06 ± 2.02	11.97 ± 1.93	11.66 ± 2.05	<0.01
RBC (10^9^/L)	3.97 ± 0.68	4.04 ± 0.69	3.99 ± 0.66	3.88 ± 0.71	<0.01
WBC (10^9^/L)	11.8 ± 8.37	12.23 ± 11.14	11.89 ± 8.19	11.17 ± 4.45	0.06
PLT (10^9^/L)	218.39 ± 82.88	221.64 ± 85.05	217.66 ± 81.46	216.48 ± 83.47	0.49
APTT (s)	31.48 ± 11.98	31.77 ± 13.45	30.88 ± 10.68	32.39 ± 12.75	0.03
PT (s)	1.22 ± 0.3	1.23 ± 0.3	1.21 ± 0.26	1.23 ± 0.35	0.15
Serum creatinine	1.1 ± 1.14	1.03 ± 0.82	1.09 ± 1.16	1.21 ± 1.36	0.01
BUN	19.18 ± 14.73	18.8 ± 14.1	17.46 ± 12.09	23.01 ± 18.91	<0.01
BUNCR	18.92 ± 7.95	18.94 ± 7.52	17.56 ± 6.98	21.6 ± 9.4	<0.01
**Comorbidities** (*n*, %)					
CCI	5.45 ± 2.86	5.36 ± 2.89	5.66 ± 2.81	5.11 ± 2.88	<0.01
Sepsis	173 (6.76)	39 (5.95)	73 (5.75)	61 (9.62)	<0.01
PVD	26 (1.02)	4 (0.61)	14 (1.10)	8 (1.26)	0.46
IVH	350 (13.68)	86 (13.13)	193 (15.20)	71 (11.20)	0.05
Hypertension	1480 (57.84)	387 (59.08)	770 (60.63)	323 (50.95)	<0.01
Liver cirrhosis	87 (3.40)	31 (4.73)	28 (2.20)	28 (4.42)	<0.01
CKD	283 (11.06)	62 (9.47)	130 (10.24)	91 (14.35)	0.01
Malignant tumors	335 (13.09)	95 (14.50)	168 (13.23)	72 (11.36)	0.24
T2DM	567 (22.16)	138 (21.07)	298 (23.46)	131 (20.66)	0.28
T1DM	28 (1.09)	9 (1.37)	10 (0.79)	9 (1.42)	0.33
Heart failure	346 (13.52)	88 (13.44)	174 (13.70)	84 (13.25)	0.96
CHD	482 (18.84)	129 (19.69)	235 (18.50)	118 (18.61)	0.81
COPD	211 (8.25)	51 (7.79)	113 (8.90)	47 (7.41)	0.48
**Treatment** (*n*, %)					
MT	1981 (77.41)	476 (72.67)	1064 (83.78)	441 (69.56)	<0.01
Vasopressor	205 (8.01)	31 (4.73)	123 (9.69)	51 (8.04)	0.10
**Outcomes** (*n*, %)					
in-hospital ACM	360 (14.07)	85 (12.98)	199 (15.67)	76 (11.99)	0.06
ICU ACM	260 (10.16)	68 (10.38)	132 (10.39)	60 (9.46)	0.80
28-day ACM	435 (17.00)	109 (16.64)	243 (19.13)	83 (13.09)	<0.01
90-day ACM	574 (22.43)	131 (20.00)	320 (25.20)	123 (19.40)	<0.01
1-year ACM	946 (36.97)	228 (34.81)	505 (39.76)	213 (33.60)	0.01

Continuous variables were presented as mean ± sd. ^a^ *p*-value for categorical variables was calculated by Chi-square test, for continuous variables was calculated by single factor ANOVA. Abbreviations: SBP, systolic blood pressure; DBP, diastolic blood pressure; MBP, mean blood pressure; SpO_2_, saturation of pulse oxygen; SOFA, sequential organ failure assessment; APS-III, acute physiology III score; SIRS, systemic inflammatory response syndrome score; SAPS-II, simplified acute physiological score II; OASIS, Oxford acute severity of illness score; GCS, Glasgow Coma Scale; Hb, hemoglobin; RBC, red blood cell; WBC, white blood cell; PLT, platelet; APTT, activated partial thromboplastin time; PT, prothrombin time; BUN, blood urea nitrogen; BUNCR, blood urea nitrogen/serum creatinine; CCI, Charlson comorbidity index; PVD, peripheral vascular disease; IVH, intraventricular hemorrhage; CKD, Chronic kidney disease; T2DM, type 2 diabetes mellitus; T1DM, type 1 diabetes mellitus; CHD, coronary heart disease; COPD, chronic obstructive pulmonary disease; MV, mechanical ventilation; ACM, all-cause mortality.

**Table 2:** Multivariate cox regression for ACM at 28 days, 90 days, and 1 year.

Categories	HR	95% CI	*p*
28-day ACM			
Group 2	Reference	-	-
Group 1	0.91	(0.72, 1.14)	0.421
Group 3	0.73	(0.57, 0.94)	0.014
90-day ACM			
Group 2	Reference	-	-
Group 1	0.80	(0.65, 0.99)	0.037
Group 3	0.80	(0.64, 0.99)	0.038
1-year ACM			
Group 2	Reference	-	-
Group 1	0.83	(0.71, 0.97)	0.021
Group 3	0.84	(0.72, 0.99)	0.037

HR, hazards ratio; CI, confidence interval. Age, gender, race, hypertension, liver cirrhosis, chronic kidney disease, diabetes, intraventricular hemorrhage and malignant tumors were adjusted for multivariate analysis.

## Data Availability

The data analyzed in this study was obtained from the Medical Information Mart for Intensive Care IV (MIMIC-IV) database (https://physionet.org/, https://doi.org/10.13026/6mm1-ek67, accessed on 11 June 2025). To access the dataset, users must be credentialed, complete the required training (CITI Data or Specimens Only Research) and sign the data use agreement for the project.

## Supplementary Materials

The following supporting information can be downloaded at: https://www.mdpi.com/article/10.3390/jcm14228141/s1, Figure S1: Trajectory model 2 (M2) identified two BUNCR trajectories: G1 (upward- downward), G2 (downward-upward). Notably, the stable-upward pattern was not identified in M2. Figure S2: Trajectory model 4 (M4) identified four BUNCR trajectories. Similarly, the stable-upward pattern (Group2) was also identified. Figure S3: Trajectory model 5 (M5) identified four BUNCR trajectories. Similarly, the stable-upward pattern (Group3) was also identified. Figure S4: Survival curves according to BUNCR trajectory groups of four-classes model (A/C/E) and five-classes model (B/D/F). Figure S5: Subgroup analysis of the association between BUNCR trajectory groups and all-cause mortality. Forest plots depict HRs with 95% CIs for 28-day (A), 90-day (B), and 1-year (C) mortality in various patient subgroups. Analyses were performed using multivariate Cox proportional hazards models adjusted for potential confounders; interaction *p*-values are shown where applicable. Table S1 Patients distributions by different trajectory model (model2/3). Table S2 Patients distributions by different trajectory model (model4/3). Table S3 Patients distributions by different trajectory model (model5/3). Table S4 AIC and BIC of different trajectory models. Table S5 Multivariate cox regression for ACM at 28 days, 90 days, and 1 year (adjusted for craniotomy). Table S6 Multivariate cox regression for ACM at 28 days, 90 days, and 1 year (adjusted for CRRT).

## Abbreviations

HS	Hemorrhagic stroke
ICU	Intensive care unit
BUNCR	Blood urea nitrogen-to-creatinine ratio
GBTM	Group-based Trajectory Modeling
MIMIC-IV	Medical Information Mart for Intensive Care-IV
ACM	All-cause mortality
ICH	Intracerebral hemorrhage
TyG-i	Triglyceride-glucose index
HRR	Hemoglobin-to-red cell distribution width ratio
BUN	Blood urea nitrogen
ICD	International Classification of Diseases
SQL	Structured Query Language (SQL)
DBP	Diastolic blood pressure
MBP	Mean blood pressure (MBP)
SpO2	Oxygen saturation measured via pulse oximetry
SOFA	Sequential Organ Failure Assessment
APS-III	Acute Physiology III score
SIRS	Systemic Inflammatory Response Syndrome
SAPS	Simplified Acute Physiology Score
OASIS	Oxford Acute Severity of Illness Score
GCS	Glasgow Coma Scale
Hb	Hemoglobin
RBC	Red blood cell count
WBC	White blood cell count
PLT	Platelet count
APTT	Activated partial thromboplastin time
PT	Prothrombin time
CCI	Charlson Comorbidity Index
PVD	Peripheral vascular disease
IVH	Intraventricular hemorrhage
CKD	Chronic-kidney disease
T2DM	Type 2 diabetes mellitus
T1DM	Type 1 diabetes mellitus
CHD	Coronary heart disease
COPD	Chronic obstructive pulmonary disease
MV	Mechanical ventilation
G1	Group 1
G2	Group 2
G3	Group 3
BIC	Bayesian Information Criterion
K-M	Kaplan–Meier
HR	Hazard ratio
CI	Confidence interval
GFR	Glomerular filtration rate
RAAS	Renin–angiotensin–aldosterone system
SCr	Serum creatinine
