# Supplementary material for: Association Between the Blood Urea Nitrogen-to-Creatinine Ratio Trajectories and Clinical Outcomes in Critically Ill Hemorrhagic Stroke Patients: Insights from MIMIC-IV Database
Source: J Clin Med. 2025 Nov 17;14(22):8141. doi: 10.3390/jcm14228141 (PMC12653523; doi:10.3390/jcm14228141)
Supplement: Supplementary file 1 [file jcm-14-08141-s001.zip › jcm-3911666-supplementary.pdf]

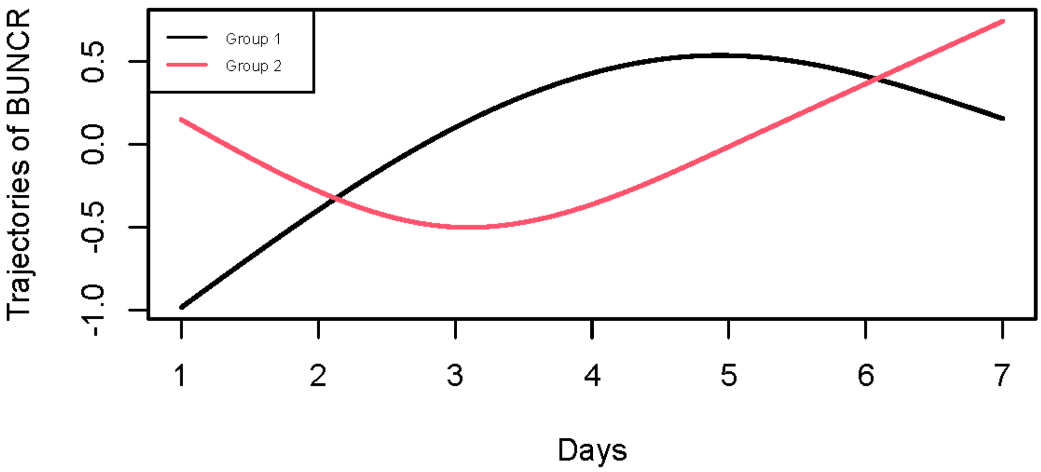

Fig.S1 Trajectory model 2 (M2) identified two BUNCR trajectories: G1 (upward- downward), G2 (downward-upward). Notably, the stable-upward pattern was not identified in M2.

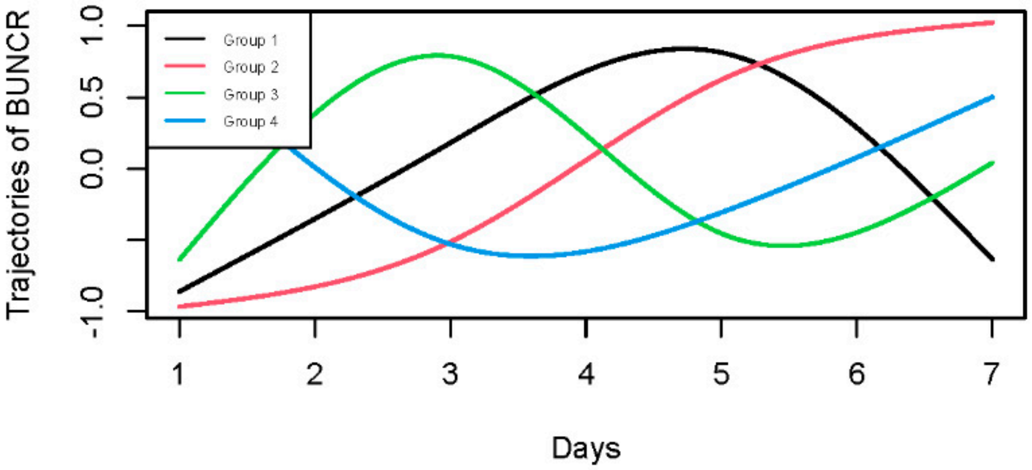

Fig.S2 Trajectory model 4 (M4) identified four BUNCR trajectories. Similarly, the stable-upward pattern (Group2) was also identified.

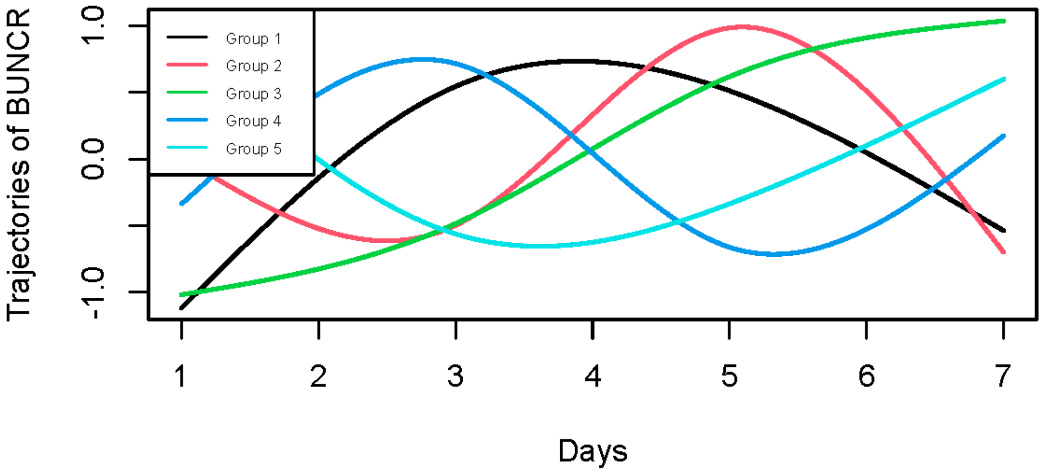

Fig.S3 Trajectory model 5 (M5) identified four BUNCR trajectories. Similarly, the stable-upward pattern (Group3) was also identified.

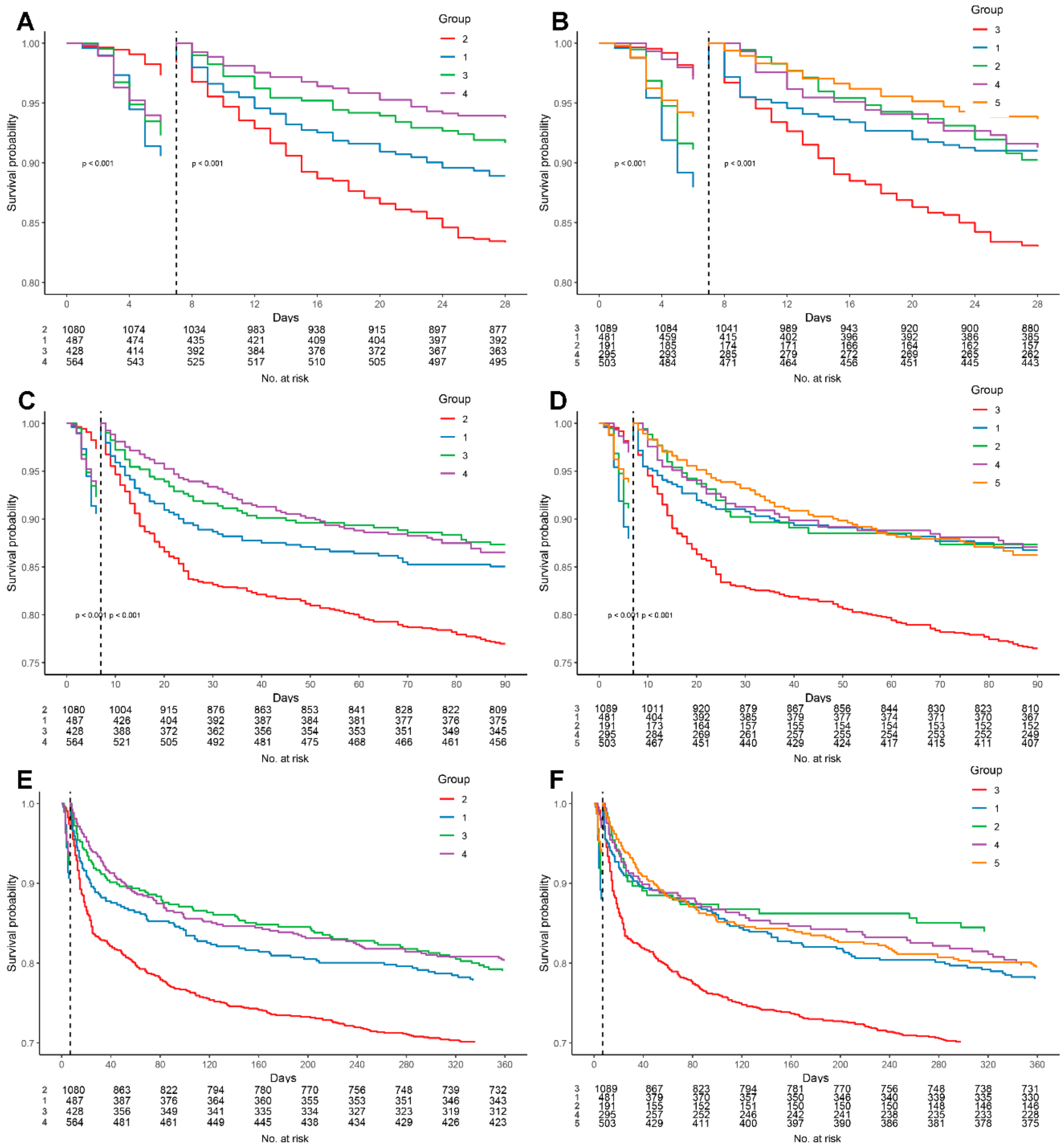

Fig.S4 Survival curves according to BUNCR trajectory groups of four-classes model (A/C/E) and five-classes model (B/D/F).

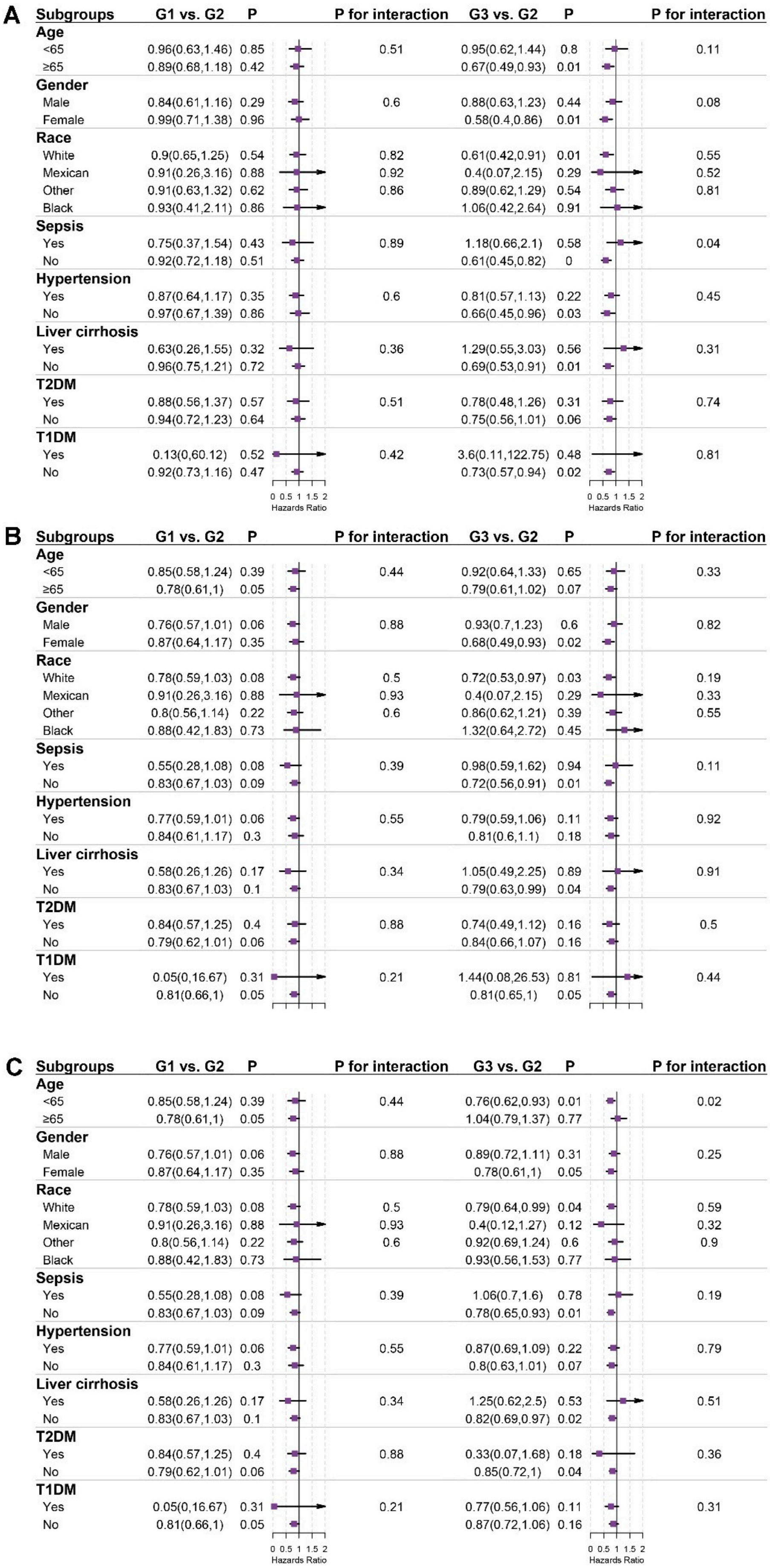
A
Subgroups G1 vs. G2 P P for interaction G3 vs. G2 P P for interaction
Age
<65 0.96(0.63,1.46) 0.85 0.51 0.95(0.62,1.44) 0.8 0.11
≥65 0.89(0.68,1.18) 0.42 0.67(0.49,0.93) 0.01
Gender
Male 0.84(0.61,1.16) 0.29 0.6 0.88(0.63,1.23) 0.44 0.08
Female 0.99(0.71,1.38) 0.96 0.58(0.4,0.86) 0.01
Race
White 0.9(0.65,1.25) 0.54 0.82 0.61(0.42,0.91) 0.01 0.55
Mexican 0.91(0.26,3.16) 0.88 0.92 0.4(0.07,2.15) 0.29 0.52
Other 0.91(0.63,1.32) 0.62 0.86 0.89(0.62,1.29) 0.54 0.81
Black 0.93(0.41,2.11) 0.86 1.06(0.42,2.64) 0.91
Sepsis
Yes 0.75(0.37,1.54) 0.43 0.89 1.18(0.66,2.1) 0.58 0.04
No 0.92(0.72,1.18) 0.51 0.61(0.45,0.82) 0
Hypertension
Yes 0.87(0.64,1.17) 0.35 0.6 0.81(0.57,1.13) 0.22 0.45
No 0.97(0.67,1.39) 0.86 0.66(0.45,0.96) 0.03
Liver cirrhosis
Yes 0.63(0.26,1.55) 0.32 0.36 1.29(0.55,3.03) 0.56 0.31
No 0.96(0.75,1.21) 0.72 0.69(0.53,0.91) 0.01
T2DM
Yes 0.88(0.56,1.37) 0.57 0.51 0.78(0.48,1.26) 0.31 0.74
No 0.94(0.72,1.23) 0.64 0.75(0.56,1.01) 0.06
T1DM
Yes 0.13(0,60.12) 0.52 0.42 3.6(0.11,122.75) 0.48 0.81
No 0.92(0.73,1.16) 0.47 0.73(0.57,0.94) 0.02
0 0.5 1 1.5 2
Hazards Ratio
0 0.5 1 1.5 2
Hazards Ratio
B
Subgroups G1 vs. G2 P P for interaction G3 vs. G2 P P for interaction
Age
<65 0.85(0.58,1.24) 0.39 0.44 0.92(0.64,1.33) 0.65 0.33
≥65 0.78(0.61,1) 0.05 0.79(0.61,1.02) 0.07
Gender
Male 0.76(0.57,1.01) 0.06 0.88 0.93(0.7,1.23) 0.6 0.82
Female 0.87(0.64,1.17) 0.35 0.68(0.49,0.93) 0.02
Race
White 0.78(0.59,1.03) 0.08 0.5 0.72(0.53,0.97) 0.03 0.19
Mexican 0.91(0.26,3.16) 0.88 0.93 0.4(0.07,2.15) 0.29 0.33
Other 0.8(0.56,1.14) 0.22 0.6 0.86(0.62,1.21) 0.39 0.55
Black 0.88(0.42,1.83) 0.73 1.32(0.64,2.72) 0.45
Sepsis
Yes 0.55(0.28,1.08) 0.08 0.39 0.98(0.59,1.62) 0.94 0.11
No 0.83(0.67,1.03) 0.09 0.72(0.56,0.91) 0.01
Hypertension
Yes 0.77(0.59,1.01) 0.06 0.55 0.79(0.59,1.06) 0.11 0.92
No 0.84(0.61,1.17) 0.3 0.81(0.6,1.1) 0.18
Liver cirrhosis
Yes 0.58(0.26,1.26) 0.17 0.34 1.05(0.49,2.25) 0.89 0.91
No 0.83(0.67,1.03) 0.1 0.79(0.63,0.99) 0.04
T2DM
Yes 0.84(0.57,1.25) 0.4 0.88 0.74(0.49,1.12) 0.16 0.5
No 0.79(0.62,1.01) 0.06 0.84(0.66,1.07) 0.16
T1DM
Yes 0.05(0,16.67) 0.31 0.21 1.44(0.08,26.53) 0.81 0.44
No 0.81(0.66,1) 0.05 0.81(0.65,1) 0.05
C
Subgroups G1 vs. G2 P P for interaction G3 vs. G2 P P for interaction
Age
<65 0.85(0.58,1.24) 0.39 0.44 0.76(0.62,0.93) 0.01 0.02
≥65 0.78(0.61,1) 0.05 1.04(0.79,1.37) 0.77
Gender
Male 0.76(0.57,1.01) 0.06 0.88 0.89(0.72,1.11) 0.31 0.25
Female 0.87(0.64,1.17) 0.35 0.78(0.61,1) 0.05
Race
White 0.78(0.59,1.03) 0.08 0.5 0.79(0.64,0.99) 0.04 0.59
Mexican 0.91(0.26,3.16) 0.88 0.93 0.4(0.12,1.27) 0.12 0.32
Other 0.8(0.56,1.14) 0.22 0.6 0.92(0.69,1.24) 0.6 0.9
Black 0.88(0.42,1.83) 0.73 0.93(0.56,1.53) 0.77
Sepsis
Yes 0.55(0.28,1.08) 0.08 0.39 1.06(0.7,1.6) 0.78 0.19
No 0.83(0.67,1.03) 0.09 0.78(0.65,0.93) 0.01
Hypertension
Yes 0.77(0.59,1.01) 0.06 0.55 0.87(0.69,1.09) 0.22 0.79
No 0.84(0.61,1.17) 0.3 0.8(0.63,1.01) 0.07
Liver cirrhosis
Yes 0.58(0.26,1.26) 0.17 0.34 1.25(0.62,2.5) 0.53 0.51
No 0.83(0.67,1.03) 0.1 0.82(0.69,0.97) 0.02
T2DM
Yes 0.84(0.57,1.25) 0.4 0.88 0.33(0.07,1.68) 0.18 0.36
No 0.79(0.62,1.01) 0.06 0.85(0.72,1) 0.04
T1DM
Yes 0.05(0,16.67) 0.31 0.21 0.77(0.56,1.06) 0.11 0.31
No 0.81(0.66,1) 0.05 0.87(0.72,1.06) 0.16
0 0.5 1 1.5 2
Hazards Ratio
0 0.5 1 1.5 2
Hazards Ratio

Fig. S5 Subgroup analysis of the association between BUNCR trajectory groups and all-cause mortality. Forest plots depict HRs with 95% CIs for 28-day (A), 90-day (B), and 1-year (C) mortality in various patient subgroups. Analyses were performed using multivariate Cox proportional hazards models adjusted for potential confounders; interaction p-values are shown where applicable.

Table.S1 Patients distributions by different trajectory model (model2/3)

Model_Group	M2_G1	M2_G2
M3_G1	626	29
M3_G2	798	472
M3_G3	6	628

Table.S2 Patients distributions by different trajectory model (model4/3)

Model_Group	M4_G1	M4_G2	M4_G3	M4_G4
M3_G1	295	2	358	0
M3_G2	183	1063	21	3
M3_G3	9	15	49	561

Table.S3 Patients distributions by different trajectory model (model5/3)

Model_Group	M5_G1	M5_G2	M5_G3	M5_G4	M5_G5
M3_G1	417	41	1	196	0
M3_G2	64	101	1080	20	5
M3_G3	0	49	8	79	498

Table.S4 AIC and BIC of different trajectory models

Model	M2	M3	M4	M5
AIC	35398.69	34409.73	33451.23	33198.51
BIC	35480.56	34509.14	33603.26	33385.63

Table.S5 Multivariate cox regression for ACM at 28 days, 90 days, and 1 year (adjusted for craniotomy)

Categories	HR	95% CI	*P*
28-day ACM			
Group 2	Refference	-	-
Group 1	0.91	(0.72, 1.14)	0.411
Group 3	0.74	(0.57, 0.95)	0.018
90-day ACM			
Group 2	Refference	-	-
Group 1	0.80	(0.65, 0.98)	0.036
Group 3	0.80	(0.65, 0.99)	0.042
1-year ACM			
Group 2	Refference	-	-
Group 1	0.83	(0.70, 0.97)	0.018
Group 3	0.88	(0.74, 1.03)	0.071

Table.S6 Multivariate cox regression for ACM at 28 days, 90 days, and 1 year (adjusted for CRRT)

Categories	HR	95% CI	*P*
28-day ACM			
Group 2	Refference	-	-
Group 1	0.95	(0.76, 1.20)	0.689
Group 3	0.70	(0.55, 0.91)	0.006
90-day ACM			
Group 2	Refference	-	-
Group 1	0.84	(0.69, 0.97)	0.044
Group 3	0.78	(0.63, 0.96)	0.021
1-year ACM			
Group 2	Refference	-	-
Group 1	0.87	(0.74, 0.98)	0.038
Group 3	0.82	(0.70, 0.97)	0.020
